# A Possible Role of Clinical Factors in Choosing the Best Treatment Modality in Cesarean Scar Pregnancy

**DOI:** 10.3390/diagnostics15080965

**Published:** 2025-04-10

**Authors:** Kwan-Heup Song, Ho-Yeon Kim, Yung-Taek Ouh, Kyung-Jin Min, Kyong-Wook Yi, Nak-Woo Lee

**Affiliations:** Department of Obstetrics and Gynecology, Korea University Medicine, Seoul 02841, Republic of Korea

**Keywords:** cesarean scar pregnancy, dilatation and curettage, transfusion

## Abstract

**Background/Objectives**: Cesarean scar pregnancy (CSP) refers to a pregnancy implanted on or within a scar from a previous cesarean birth. This study aims to evaluate the treatment strategies for CSP conducted at a single center and analyze the predictive markers of a single procedure without complications. **Methods**: A retrospective study was performed on CSP patients who received treatment at Korea University Ansan Hospital from November 2002 to December 2022. The clinical characteristics of CSP and the occurrence of complications based on treatment methods were investigated. **Results**: A total of 128 patients were included in the study. Among them, 84 patients (65.6%) underwent dilatation and curettage (D&C) only, 12 patients (9.4%) received a combination of methotrexate and D&C, 21 received D&C with Foley catheter ballooning (15.7%), and 11 patients (9%) underwent invasive procedures such as uterine artery embolization, hysterectomy, and open resection. As gestational age increased, the likelihood of opting for invasive procedures (aOR = 3.52, 95% CI 1.74–10.14, *p* = 0.003) also increased. A total of 84 patients (65.6%) were successfully treated with D&C without transfusion, and it was found that early gestational age and sonographic hypervascularity played a significant role. **Conclusions**: Early gestational age, younger maternal age, and sonographic hypervascularity were independently associated with favorable treatment without adjuvant therapy and transfusion. Therefore, for a safe and bloodless approach to treatment in cases of CSP, it is advisable to promptly diagnose this condition as early as possible.

## 1. Introduction

The rate of cesarean scar pregnancy (CSP) is growing as the rate of cesarean section increases [1,2]. It is a serious form of ectopic pregnancy where the conceptual tissue implants within the disrupted myometrial scar tissue of a previous cesarean section. Although underreported and underdiagnosed, the incidence ranges from 1 in 1800 to 1 in 2656 [3]. This condition poses significant risks due to its potential for severe complications, including placenta accreta spectrum, uterine rupture, massive hemorrhage, hysterectomy, and subsequent infertility [4,5]. The corresponding rise in the incidence of CSP has made it an essential focus of urgent medical care.

The diagnosis of CSP starts with patient history taking whether the patients have a history of cesarean section. When there is a suspicion of pregnancy in these women, early ultrasound scan as early as within 6 weeks should be performed to allow for an early diagnosis. Gestational sac location determines CSP in the first trimester with all pregnancies implanted in or in close contact with the niche or isthmocele—defined by a Delphi consensus as an indentation of at least 2 mm at the site of the CS scar [6]. Other terms that aid in the diagnosis of CSP include a crossover sign or low-implantation sign [7,8]. As the gestational age advances, this sac progressively grows external uterine contour or serosal line toward the bladder and abdominal cavity, predisposing to rupture or progression to placenta adhesion [9]. The cases with endogenic growth of the sac are associated with less severe morbidity in early pregnancy but a delayed diagnosis [10].

The management of CSP is particularly challenging, as it requires careful consideration of both maternal safety and future fertility. Recommended treatment usually takes place in the first trimester. Various treatment modalities have been employed to address CSP [11,12], including nonsurgical approaches, such as expectant management, systemic and local administration of embryocidal agents such as methotrexate (MTX) [13,14], high-intensity focused ultrasound therapy [15], and pelvic artery embolization; surgical interventions, like dilation and curettage (D&C) with or without the use of Foley ballooning and hysteroscopic resection; and further invasive surgical procedures, such as hysterectomy, or open or laparoscopic resection [16]. All these methods are utilized independently or combined depending on the clinical scenario. But the optimal treatment strategy for CSP without complications and without invasive treatment remains a subject of ongoing controversy.

The clinical characteristics of CSP, such as gestational age at diagnosis, the thickness of the myometrial scar, the presence of fetal cardiac activity, and the size and location of the gestational sac, can significantly influence the choice of treatment and its outcomes [17,18,19,20]. For instance, cases with a thin myometrial layer overlying the gestational sac or those diagnosed later in pregnancy may require more aggressive management to prevent life-threatening complications [21]. Conversely, early diagnosis and favorable clinical characteristics might allow for successful conservative management with lower morbidity.

Despite the critical role these clinical characteristics play, there is a scarcity of objective data examining how they influence treatment outcomes across different management strategies. This study aimed to analyze the clinical utility of various factors for treatment efficacy and safety and provide a treatment for CSP which results in favorable patient outcomes without complications.

## 2. Materials and Methods

### 2.1. Study Design and Setting

This retrospective study was conducted by reviewing patient charts and ultrasound imaging at Korea University Ansan Hospital, covering the period from 1 November 2002 to 31 December 2022. Blind sonographic evaluation in this field was performed independently by two experts without knowledge of clinical and pathologic outcomes. We excluded patients with other types of ectopic pregnancy or cesarean scar pregnancy (ICD 10th Revision codes in the Hospital Discharge Register, O00.8 and O00.9); with cervical pregnancy; with hemoperitoneum with no ectopic mass; with abdominal, adnexal, or cornual pregnancy; lost at follow-up; or with conservative management. The diagnosis of CSP was made ultrasonographically based on the criteria of Society of Maternal–Fetal Medicine Consult Series #63: (1) an empty uterine cavity and endocervix; (2) placenta, gestational sac, or both embedded in the hysterotomy scar; (3) a triangular (at ≤8 weeks of gestation) or rounded or oval (at >8 weeks of gestation) gestational sac that fills the scar “niche” (the shallow area representing a healed hysterotomy site); (4) a thin (1–3 mm) or absent myometrial layer between the gestational sac and bladder; (5) a prominent or rich vascular pattern at or in the area of a cesarean scar; (6) an embryonic or fetal pole, yolk sac, or both, with or without fetal cardiac activity [1]. This study was approved by the institutional review board of Korea University Ansan Hospital with a waiver for informed consent.

### 2.2. Patient Selection

Demographic and clinical factors including age, gestational age, number of cesarean sections, and the rate of transfusion were assessed. Gestational age was confirmed by the first day of the last menstrual period or ultrasonography measurements. Serum beta hCG, the size of the gestational sac, the size of conceptual tissue in the case of invisible gestational sac, the presence of fetal heartbeat (FHB), crown–rump length, and residual myometrial thickness (RMT) were reviewed. Patients were categorized into the following treatment groups: D&C, D&C combined with systemic or local administration of methotrexate (MTX), D&C combined with Foley ballooning, and invasive procedures including D&C with pelvic artery embolization, hysterectomy, or open resection.

Systemic MTX was administered before D&C at a dosage of 50 mg/m^2^. The dosage of local MTX administration was 10–12.5 mg, which was transcervically injected into the gestational sac under transvaginal ultrasound guidance. All MTX treatments were accompanied by D&C before or after treatment. Foley ballooning in the uterine cavity for mechanical hemostasis was an adjuvant therapy after D&C in the case where profuse bleeding occurred or was expected. Pelvic artery embolization, hysterectomy, or open resection for direct bleeding control were performed in the case of profuse bleeding.

### 2.3. Imaging Acquisition

**Figure 1:**
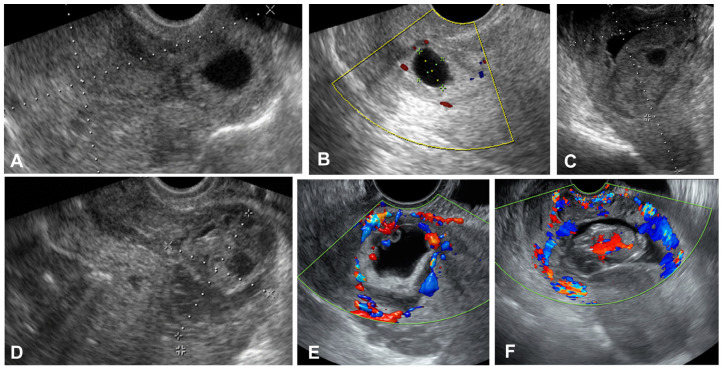
Ultrasonographic images of cesarean scar pregnancy. (**A**) Low implantation and bulging of gestational sac in lower uterine segment. (**B**) Low implantation of small blood flow around the gestational sac with empty uterine cavity and cervical canal. (**C**,**D**) Heterogenous mixed placental tissue bulging in lower uterine segment. (**E**,**F**) Color Doppler imaging showing blood flow around the gestational sac at the site of implantation suggestive of “hypervascularity”.

All ultrasound images at admission were retrieved from medical records. All stored images demonstrated the gestational sac or conceptual tissues within a cesarean section scar (Figure 1). Grayscale and/or color Doppler transvaginal ultrasound (Voluson Expert 22, E10 and E8 (GE, Tiefenbach, Zipt, Austria), Medison Accuvix XQ (Seoul, Republic of Korea)) was used, and the diagnosis was determined by the Society for Maternal–Fetal Medicine Consult Series criteria and the modified Delphi method [1,6]. We defined “hypervascularity” in cases where there was a profuse vascular pattern around cesarean scar pregnancy in color Doppler images (Figure 1E,F). In the absence of color Doppler images, it was not possible to analyze the presence of hypervascularity.

**Figure 2:**
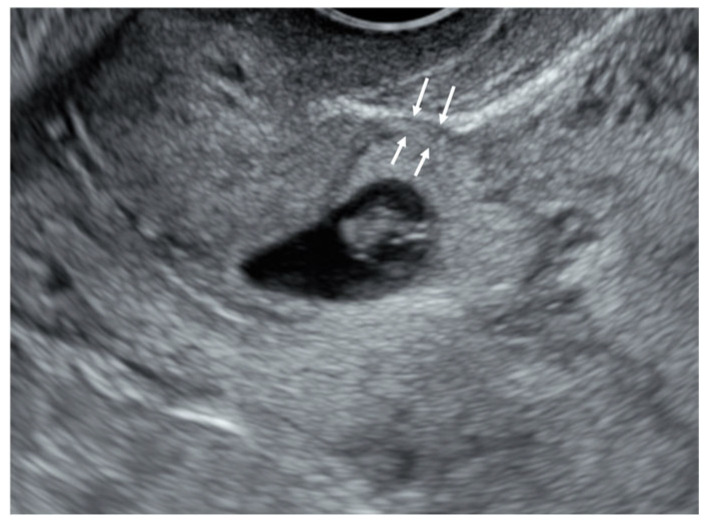
Measurement of residual myometrial thickness (RMT) indicated by arrows.

Residual myometrial thickness (RMT), or the thinnest part of the lower segment of the myometrium where the GS is situated, was defined in a sagittal section as the shortest length between the outer edge nearest to the myometrium of the gestational sac and the uterine serosa (Figure 2) [22]. All patients were confirmed to be positive to the urine human chorionic gonadotropin test or serum beta hCG (≥5 mIU/mL) or conceptual tissue on the pathologic report.

### 2.4. Statistical Analysis

Data were analyzed by using SPSS software, version 20.0 (SPSS Inc., Chicago, IL, USA). Statistical significance was determined with a *p*-value threshold of less than 0.05. The analysis included the Shapiro–Wilk normality test, Chi-square test, Student’s *t*-test, Fisher’s exact test, the non-parametric Mann–Whitney U test, and ANOVA. Additionally, logistic regression analysis was performed to identify factors associated with outcomes after adjustment for confounding factors. Receiver operating characteristics curves were calculated to evaluate the accuracy of factors associated with successful first-line treatment with adjunctive therapy.

## 3. Results

A total of 133 patients diagnosed with CSP were analyzed. Five cases were lost at follow-up. Among the remaining patients, 84 underwent D&C, 12 underwent D&C with systemic or local MTX injection, 21 underwent D&C with Foley ballooning, and 11 underwent invasive procedures. The rate of having one prior cesarean section was 41.4%, while the rate of having two prior C-sections was 49.6%. The remaining patients had more than three prior cesarean sections. There were four cases of hysterectomy in the invasive procedure group, with three women having had two previous cesarean sections and one woman having had one cesarean section. There were no differences in maternal age, serum beta hCG at admission, the number of cesarean sections, the rate of fetal heartbeat and the residual myometrial thickness in the ultrasound (Table 1).

The gestational age was higher (*p* < 0.001) and the size of the gestational sac and CRL were significantly larger (*p* < 0.001) in the invasive treatment group compared with the non-invasive group. Additionally, this group required the highest number of blood transfusions. The rate of sonographic hypervascularity near the gestational sac or conceptual mass was significantly high in the invasive treatment group. Hypervascularity was the highest in the invasive group, followed by D&C Foley ballooning, D&C with methotrexate, and D&C only.

In the group treated with D&C combined with Foley ballooning, higher beta hCG levels and larger g-sac sizes were observed, with 47.6% of patients requiring blood transfusions and 52.9% of fetal heartbeat being observed, which was significantly more frequent compared with the group treated with D&C alone and D&C with methotrexate.

Multiple logistic analysis was utilized to assess the risk factors for transfusion and invasive procedures. In cases with transfusion, none of the risk factors were independently associated after adjustment (Table 2).

In cases with invasive procedures, the gestational age was 3.5 times higher (*p* = 0.003) after adjustment for maternal age, fetal heartbeat, and the number of cesarean sections (Table 3).

To determine the safe factors associated with CSP treatment, we performed a logistic analysis for D&C without transfusion. The gestational age was significantly lower, about 63.1%, and the rate of sonographic hypervascularity was much lower, about 84.4% (Table 4). Residual myometrial thickness, fetal heartbeat, and the number of cesarean sections were not associated D&C without transfusion.

**Figure 3:**
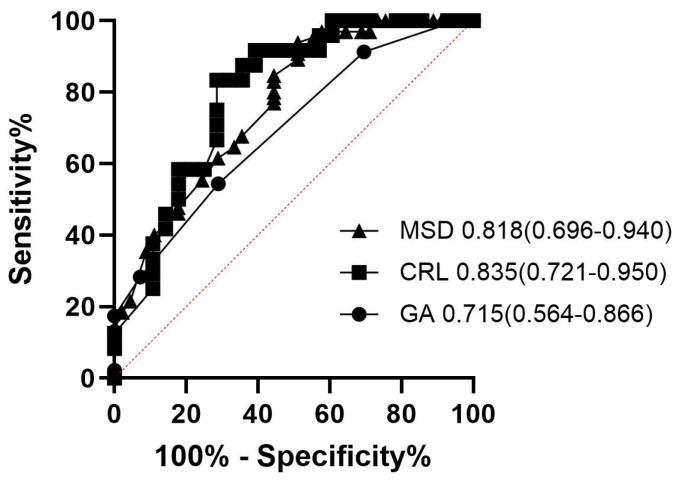
Receiver operating characteristic curves showing diagnostic performance of gestational age, crown–rump length, and mean gestational sac size for dilatation and curettage without any adjunctive therapy or transfusion.

The ROC analysis results for the prediction of successful first-line treatment without adjunctive therapy or transfusion were 0.715 (95% CI 0.564–0.866 *p* < 0.001), with an estimated best cutoff range of 6.5 gestational weeks, a sensitivity of 70.4%, and a specificity of 61.1%; 0.835 (95% CI 0.721–0.950), with an estimated best cutoff of 0.52 cm crown–rump length, a sensitivity of 74.1%, and a specificity of 83.3%; and 0.818 (95% CI 0.696–0.940), with an estimated best cutoff of 2.45 cm mean sac diameter, a sensitivity of 74.1%, and a specificity of 83.3% (Figure 3).

## 4. Discussion

The rate of cesarean section is more than half of deliveries in South Korea, and more than one-third was reported in the United States in 2021 [23,24]. Therefore, with the increasing number of cesarean sections, the rise in CSP is an inevitable reality, and preparedness for this condition is essential. The findings from this study show that maternal age, serum beta hCG at admission, the number of cesarean sections, and RMT in the ultrasound were not different among treatment modalities in CSP. Instead, a higher gestational age was an independent risk factor for stratifying invasive procedures. In contrast, a lower gestational age and the rate of hypervascularity were independently associated with dilatation and curettage without any adjunctive therapy or transfusion.

Recently, there has been growing evidence that supports expectant management of CSP. The livebirth rate is reported to be quite high in expectant management, but catastrophic events such as severe placenta accreta spectrum can occur during expectant management [5,25]. Therefore, evidence-based guidelines strongly suggest active or surgical management instead of expectant management [1]. The success rate of active management was reported to be ~97.5% [26]. Although an optimal treatment method has not been determined yet, previous studies recommend methotrexate injection with or without adjunctive therapy, dilatation and curettage, hysteroscopy, laparoscopy, open resection, and pelvic arterial embolization either as an adjunctive treatment or as a treatment, and finally, hysterectomy [27,28]. One meta-analysis reported 31 different treatment approaches with complication rates higher than 40% [29]. Thus, there have been a small number of cases and scanty RCTs in the evaluation of successful treatment in CSP due to different treatment selection by various experts with preferences.

The early detection of CSP would allow for prompt referral to a tertiary center with experts and facilities with resources such as adequate transfusion. In cases where future fertility is desired, the early and precise diagnosis with appropriate treatment is essential. A previous meta-analysis comprising 37 studies showed that an early, first-trimester diagnosis at less than 9 weeks of gestation decreases adverse maternal outcomes [7]. However, this study did not mention various treatment strategies and how these strategies influenced maternal outcomes.

A previous report on RMT presented that less than 2 mm in RMT contributes to the occurrence of higher complications in CSP or ongoing pregnancy [6], but our results showed no differences in RMT among various treatments. This might imply that the treatment outcome might not be affected by RMT in CSP cases with active management. In addition, a previous evaluation of RMT demonstrated inconsistent results and mainly focused on uterine rupture [30]. The difference between our results and previous works might be attributed to varied RMT, which suggests the low implantation of the gestational sac without any niche or isthmocele [31]. Since our study is retrospective, it is challenging to confirm the presence of niches or isthmoceles. However, most of our research pertains to the first trimester of pregnancy, and we would like to emphasize that increased vascular development leading to increased blood flow rather than the thinning of RMT may be an important factor in achieving treatment without complications. Another study to determine risk factors for intraoperative bleeding demonstrated anterior myometrial thickness at the scar and the diameter of the gestational sac. The study developed a new classification model of CSP to predict outcomes based on anterior myometrial thickness and gestational sac diameter, and the success rate was 97.5%. However, the study did not include the size of the crown–rump length or sonographic hypervascularity, the treatment options are limited, and the standard of intraoperative bleeding of less than 300 mL might be underestimated for CSP, or most of the cases were non-severe [26]. An independent value of RMT in CSP should be considered in future prospective studies.

Hypervascularity or increased vascular formation around the gestational sac is known as abnormal trophoblastic flow and vascular complications. This happens due to the loss of the normal uterine structure in the scar followed by direct contact of large-diameter uterine arteries in placental tissue causing arteriovenous shunts [32,33]. The results of this pathophysiology is more hypervascularity around the gestational sac as the gestational age advances. The standardization of hypervascularity in Doppler ultrasound is not established yet, but the gynecologic group described the score of blood flow at Doppler imaging as 1 to 4. The adaption of this scoring system was utilized in a CSP study that showed a higher score in CSP compared with normal pregnancy [34]. This suggests abnormal vascular remodeling in CSP from early implantation. Our study further demonstrated that cases with less apparent hypervascularity provide evidence that reduced hypervascularity is associated with less bleeding and fewer complications.

In cases of hemorrhage before or during procedures, transferring a patient to a tertiary hospital can be highly risky. Excessive bleeding may occur during transportation, potentially endangering the patient’s life. Therefore, if the treatment guideline for CSP is established to allow for simple D&C alone, safe treatment could also be considered at primary or secondary care hospitals. This study is expected to help provide such a basis. A previous study analyzed 195 women with CSP, and 41.5% were undiagnosed in primary and secondary hospitals, which was followed by a higher rate of complications [35]. If the gestational age is low and there is no sonographic hypervascularity, treatment at non-tertiary hospitals could be considered rather than transfer. However, preparation for acute bleeding is indispensable before procedures.

### Strengths and Limitations

This study comprises more than 100 CSP cases, which is one of the largest retrospective populations in consideration of treatment. Ultrasound professionals blindly reviewed ultrasound images to determine whether the case was true CSP or not. All the low-implantation normal pregnancy, cervical pregnancy, and incomplete abortion cases were excluded from the analyses. However, there are major pitfalls. First, the retrospective nature of this study is a major limitation. Although the treatment of CSP has evolved over the past 20 years, this study did not employ HIFU or hysteroscopic surgery. Instead, its significance lies in evaluating whether CSP can be treated simply, without invasive procedures and without bleeding. Second, predictive ultrasonographic markers, including hypervascularity and growth pattern, were limited in this study due to a lack of imaging records. The standardization of ultrasonographic parameters for CSP has not been established yet; therefore, hypervascularity might be subjective. In addition, blood loss count, which is a determinant factor, was not recorded, so it could not be analyzed in this study.

## 5. Conclusions

The treatment goal for CSP is to preserve maternal health and fertility. For women with more than one cesarean section, ultrasound should be thoroughly performed in the case of intrauterine pregnancy as early as possible. After the diagnosis of CSP, if findings such as gestational age ≥ 6.5 weeks, a gestational sac ≥ 24.5 mm, a crown–rump length ≥ 5.15 mm, or sonographic hypervascularity are present, adjunctive therapy, including transfusion, balloon tamponade, or invasive procedures, might be required during treatment. The prompt referral of these patients with high-risk factors is needed when resources are inadequate.

## Figures and Tables

**Table 1:** Maternal characteristics, ultrasound findings, and pregnancy outcomes.

	D&C (*n* = 84)	D&C + MTX (*n* = 12)	D&C + Foley Ballooning (*n* = 21)	Invasive Treatment ^a^ (*n* = 11)	*p*-Value
Age (years)	34.9 ± 4.9	37.8 ± 4.7	35.1 ± 4.7	35.4 ± 5.0	0.333
Gestational age (weeks)	6.1 ± 1.0	6.9 ± 1.3	7.0 ± 1.4	8.1 ± 2.3	<0.001
Beta hCG	28,940 ± 116,770	18,476 ± 17,864	43,868 ± 38,287	32,754 ± 31,978	0.944
Mean gestational sac diameter (cm)	1.6 ± 0.8	2.4 ± 1.3	3.2 ± 1.1	4.7 ± 2.6	<0.001
CRL (cm)	0.4 ± 0.3	1.1 ± 1.2	1.1 ± 0.8	2.6 ± 2.2	<0.001
RMT (cm)	0.3 ± 0.1	0.38 ± 0.17	0.26 ± 0.11	0.21 ± 0.11	0.033
Number of csec	1.6 ± 0.6	1.8 ± 0.7	1.8 ± 0.5	1.6 ± 0.7	0.746
FHB (%)	30.7	36.4	52.9	62.5	0.146
Transfusion (%)	14.5	25	47.6	72.7	<0.001
Hypervascularity (%)	14	37.5	75	100	<0.001

D&C: dilatation and curettage; MTX: methotrexate; hCG: human chorionic gonadotropin; CRL: crown–rump length; RMT: residual myometrial thickness; csec: cesarean section; FHB: fetal heartbeat. ^a^ Invasive treatment: pelvic artery embolization, hysterectomy, or open or laparoscopic resection.

**Table 2:** Logistic regression analysis for transfusion in cesarean scar pregnancy.

	OR	95% CI	*p*-Value	aOR	95% CI	*p*-Value
Gestational age	1.311	0.962–1.804	0.086	1.490	0.930–2.557	0.114
RMT	0.051	0.001–1.284	0.089	0.064	0.001–7.494	0.287
Number of csec	0.917	0.471–1.756	0.794	1.068	0.366–3.016	0.900
FHB	1.344	0.539–3.286	0.517	1.973	0.519–7.621	0.314
Hypervascularity	3.152	1.171–8.743	0.024	1.298	0.249–6.218	0.747

RMT: residual myometrial thickness; csec: cesarean section; FHB: fetal heartbeat.

**Table 3:** Logistic regression analysis for invasive procedures in cesarean scar pregnancy.

	OR	95% CI	*p*-Value	aOR	95% CI	*p*-Value
Gestational age	2.115	1.345–3.583	<0.001	3.524	1.740–10.14	0.003
FHB	5.156	1.05–37.37	0.058	11.39	1.13–360.6	0.076
Number of csec	0.936	0.320–2.556	0.899	1.249	0.186–8.083	0.807
Age	1.008	0.888–1.151	0.899	1.296	0.978–1.885	0.113

FHB: fetal heartbeat; csec: cesarean section.

**Table 4:** Logistic regression analysis for D&C without transfusion in cesarean scar pregnancy.

	OR	95%CI	*p*-Value	aOR	95% CI	*p*-Value
Gestational age	0.495	0.324–0.706	<0.001	0.388	0.158–0.774	0.018
RMT	5.057	0.366–84.26	0.238	0.037	0.001–3.925	0.168
Number of csec	0.817	0.456–1.455	0.494	0.472	0.154–1.303	0.159
Hypervascularity	0.125	0.039–0.349	<0.001	0.156	0.029–0.692	0.048
FHB	0.563	0.256–1.227	0.149	0.400	0.095–1.629	0.199

D&C: dilatation and curettage; RMT: residual myometrial thickness; csec: cesarean section; FHB: fetal heartbeat.

## Data Availability

The datasets used and analyzed during the current study are available from the corresponding author upon reasonable request.

## Abbreviations

CSP	cesarean scar pregnancy
hCG	human chorionic gonadotropin
D&C	dilatation and curettage
csec	cesarean section
FHB	fetal heartbeat
MTX	methotrexate
RMT	residual myometrial thickness
